# Physical-intellectual badminton teaching intervention for children with intellectual disabilities

**DOI:** 10.3389/fpsyg.2025.1445620

**Published:** 2025-03-11

**Authors:** Yu Wang, Delai Zhou, Chuang Liu, Lingyu Long, Gong Cheng

**Affiliations:** ^1^Department of Theory and Methodology of Sport and Physical Education, Moscow State University of Sport and Tourism, Moscow, Russia; ^2^Department of Physical Education, Harbin Far East Institute of Technology, Limin Development Zone, Harbin, China

**Keywords:** intellectual, disability, “physical intelligence” fusion, cognitive ability, physical quality, basic movement skills

## Abstract

**Objective:**

To promote the healthy development of adolescents with intellectual disabilities, this study uses badminton to combine sports intervention with cognitive intervention to explore the content of sports teaching and cognitive intervention programs suitable for the learning of students with intellectual disabilities.

**Methods:**

This research selected 26 mildly mentally disabled students in special education schools (age: 14.5 ± 0.8 years old), the subjects were randomly assigned to three groups by the digital randomization method, which badminton physical intelligence group (BSI), badminton group (BS) and control group (CON), with BSI conducting “physical intelligence” integration badminton intervention, and BS conducting badminton intervention, the intervention cycle was 12 weeks, with 3 teaching sessions per week, each session lasted for 40 min. The subjects’ cognitive abilities and basic motor skills were analyzed.

**Results:**

The results showed that BSI had highly significant differences in all cognitive ability test items (*p* < 0.01); BS had significant differences only in visual attention, visual memory, and motor imitation (*p* < 0.05). The results of incremental changes between groups before and after the intervention showed that BSI compared with CON had significant differences in all aspects except in object constancy (*p* < 0.05); BS compared with CON had higher incremental means than CON in visual attention, visual memory, and movement imitation, with significant differences (*p* < 0.05); BSI compared with BS had significant differences in all aspects except in object constancy and visual memory aspects, there is a significant difference (*p* < 0.05).

**Conclusion:**

The “Body-Smart Integration” badminton intervention can improve the cognitive ability of students with intellectual disabilities in visual, auditory, imitation, concept learning, object permanence, etc., and the effect of improving the cognitive ability of students with intellectual disabilities is better than that of the badminton group and the control group.

## 1 Introduction

In special education today, the education and development of students with intellectual disabilities have become increasingly prominent (Morgan et al., 2015). This is caused by environmental or genetic influence of the patient’s genetic (gene) system abnormal production of a mental development before birth, during birth or early life stages due to various reasons are hindered and stop the development of comprehensive symptoms. Mildly retarded people have poor learning ability and discounted ability to adapt to life, mainly in the inability to understand problems correctly, unable to think flexibly, and slow to solve problems (Prokhorenko and Makarov, 2022). Owing to congenital or acquired deficiencies, they generally face multiple challenges, such as poor physical fitness, low levels of cognitive ability, and lagging levels of motor skill development, which not only seriously affects the quality of their daily lives but also dramatically restricts their ability to integrate into society (Kaya and Cavkaytar, 2021). With the increased attention paid to the cause of special education and the introduction of a series of policies and regulations, how to effectively promote the healthy physical and mental development of students with intellectual disabilities and improve their social adaptability has become an urgent issue in the field of special education (Maenner et al., 2021; Baio et al., 2018).

Faced with the problem of lagging physical and mental development of students with intellectual disabilities, researchers have conducted numerous explorations and studies. Previous studies have shown that physical education and sports interventions have significant effects in enhancing the physical fitness and motor skills of students with intellectual disabilities, while cognitive interventions help to improve their cognitive abilities (Carraro and Gobbi, 2014; Stanish and Temple, 2012; Ginis et al., 2021; Robertson et al., 2018; Jacinto et al., 2021).

As a popular and exciting sport, badminton can not only effectively exercise physical fitness and improve motor skills but also cultivate quality and promote interpersonal skills, which positively affects the physical and mental health development of students with intellectual disabilities (Latino et al., 2018). Therefore, this study combines badminton with cognitive training and explores the positive effects on the physical and mental development of students with intellectual disabilities by integrating the “physical and intellectual” teaching intervention model.

Firstly, a “physical and intellectual” integrated badminton teaching intervention model for students with intellectual disabilities will provide new ideas and methods for physical education teaching in special schools (Trollor et al., 2018). This model will consider the characteristics of students with intellectual disabilities, fully consider their physical conditions and cognitive levels, and develop a targeted teaching program (Page and Cannella-Malone, 2019).

Secondly, through empirical research, we verified the positive effects of badminton’s “physical and intellectual” integration on the physical fitness, motor skills, and cognitive abilities of students with intellectual disabilities (Fu et al., 2024). By comparing the data before and after the experiment, we can evaluate the effect of the intervention and provide a scientific basis for the Practice of special education.

Finally, the badminton teaching intervention program and the specific implementation content are formulated to suit the students with intellectual disabilities by considering their characteristics. This will help improve the teaching quality of special schools and promote the overall development of students with intellectual disabilities.

Therefore, this study aims to explore the positive impact on the physical and mental development of students with intellectual disabilities through the integration of badminton’s “physical and intellectual” pedagogical interventions to contribute to developing the cause of special education. By constructing a teaching intervention model suitable for students with intellectual disabilities, verifying its effectiveness, and developing corresponding implementation contents, this study will provide new ideas and methods for the Practice of special education and promote the comprehensive development of students with intellectual disabilities.

## 2 Materials and methods

### 2.1 Research population

Twenty-six mildly intellectually disabled students aged 13–16 were selected as subjects for this experiment. Through the test results of basic motor skills, physical fitness, and cognitive ability in the pre-experimental period, the students were grouped into badminton physical and intellectual group (BSI, *n* = 9), badminton group (BS, *n* = 8), and control group (CON, *n* = 8). The badminton physical and intellectual group was provided with 12 weeks of physical-intellectual integration of badminton teaching and learning interventions, the badminton group was provided with 12 weeks of badminton teaching and learning interventions, and the control group does not provide any instructional or specifically organized exercise interventions. In the other two badminton teaching groups, except for the specially organized badminton intervention program, no organized sports (such as sports classes, sports games, etc.) are provided, but the normal physical activities of the subjects are not restricted. Any intervention was carried out, with one 40-min classroom session thrice weekly. The relevant local ethics committee approved the study, approval no: SY-ZQ-2024-122. This study conducted the grouping of subjects in accordance with the method of randomized controlled trials. In brief, each subject was numbered using a random number generator on a computer and randomly assigned to one of the three groups. The calculation method of sample size of subjects referred to previous studies and combined with the characteristics of this study, SAS Power and Sample Size application (PSS) method was used for calculation, and 8 subjects in each group were finally selected as the minimum suitable number.

Inclusion Criteria: 1. Mildly mentally disabled students as the subject group. 2. Subjects were able to participate in sports. 3. Subjects had basic understanding and communication skills.

Exclusion Criteria: 1. Subjects could not participate in normal sports activities due to physical disabilities or illnesses. 2. Subjects had aggressive behaviors in daily activities. 3. Subjects had difficulties in communicating with the teachers.

### 2.2 Exercise intervention program

The content of this experiment is divided into the badminton group intervention program and the badminton Rong Zhi group intervention program; the intervention content of the two groups is the same; the preparation part is through the traditional warm-up activities so that the students can be fully warmed up, to prevent the emergence of sports injuries. The end part was based on finishing and relaxation, and the content was the same. Physical fitness exercises were the same in both groups and were carried out utilizing small games or competitions; according to the developmental characteristics of the individual qualities, speed, agility, coordination, and balance qualities were practiced in the first half of the lesson, and strength, endurance, and flexibility qualities were practiced in the second half of the lesson. Based on the intervention period selected by a large number of previous studies and the semester period of the normal population in this age group, we chose a 12-week (3-month) intervention duration in this study.

The difference in the intervention content was in the basic part, where the badminton integration group incorporated cognitive interventions, such as auditory ability, visual ability, conceptual learning, and imitation ability, on top of badminton technical exercises to improve cognitive abilities.

The details of the weekly training components of the sports intervention program are shown in Table 1, while the details of the “plus wisdom” program for cognitive skills are shown in Table 2.

**Table 1:** Details of the content of badminton teaching.

Time	BS group	BSI group
Week 1	① Handicap	① Forward hand ball + auditory attention/visual attention/movement imitation
	② Handicap between rows	② Marching forehand + concrete concept learning/auditory attention/object eternity
	③ Inter-march forehand and backhand	③ Marching forehand and backhand turnover + abstract concept learning/auditory attention/visual attention/verbal imitation
Week 2	①Forehand shot	①Forehand turnover+motion imitation/visual attention/visual memory/object permanence
	②Forward and backhand turnover in the line of scrimmage	② inter-march forehand and backhand turnover + abstract concept learning/auditory attention/visual attention/language Imitation
Week 3	①Review ballistic exercises	①Ballistic exercises + visual attention/auditory attention/verbal imitation/visual memory/auditory memory/abstract concept learning
Week 4	①Backhand serve	①Backhand serve + movement imitation/visual attention/visual memory/object permanence/abstract concept learning
	②Backhand serve at the net	② Backhand serve at the net + visual attention/concrete concept learning/verbal imitation
Week 5	①Sphericality exercise	①Sphericality + concrete concept learning/movement imitation/language imitation/object eternity/abstract concept learning
	②Forehand high ball	②Forehand serve + movement imitation/visual attention/visual memory/object permanence/abstract concept learning
	③Backhand serve at the net	③Backhand serve over the net + visual attention/concrete concept learning/verbal imitation
Week 6	①Cross-step exercise	①Cross-step exercise + visual memory/auditory memory
	②Cross-step exercises	②Cross-step exercise + concrete concept learning/abstract concept learning
	③Stepping exercise	③Stomping exercises + object eternity/abstract concept learning
Week 7	①Forehand serve	①Forehand serve + verbal imitation/visual attention/object eternity
	②Pad exercise	②Pad exercise + movement imitation/concrete concept learning
	③Steps and turns	③Stomping and turning exercises + auditory memory/visual memory
	④Step combination exercise	④Pace combination exercises + movement imitation/auditory attention
Week 8	①Ballistic exercise	①Ballistic exercises + abstract concept learning/object constancy
	②Serve exercise	② Serving practice + verbal imitation/movement imitation
Week 9	①Forehand shot	①Forehand shot+motion imitation/visual memory/concrete concept learning
	②Backhand pick	②Forehand pick + visual attention/abstract concept learning/object eternity
Week 10	①Forehand shot	①Forehand shot + object eternity/abstract concept learning/visual memory
	②Handicap	②Backhand pick + visual attention/visual memory/movement imitation
	③Hand hook	③Forehand hook + verbal imitation/auditory memory/auditory memory
Week 11	①Ball practice	①Ball Practice + visual attention/auditory attention/verbal imitation/
	②Hitting practice	② batting practice + concrete concept learning/abstract concept learning/object eternity
	③Pair batting practice	③Double batting practice + verbal imitation/movement imitation/visual attention
Week 12	①Pace + strike combination exercise	①Pace + striking combination drill + auditory memory/concrete concept learning/visual attention
	②Hitting practice	② Hitting practice +/object eternal/auditory attention/auditory memory/concrete concept learning/abstract concept learning
	③1 V1 match	

**Table 2:** Details of the content of the “PlusSmart” cognitive training program.

Exercise content	Cognitive intervention	Plus intellectual methods
Invert the ball	Visual attention	Balloons were used as a substitute for the pre-badminton practice, and during the badminton bobble, the teacher held three colored badminton balls and passed by the practicing students and asked for the number or colors they had seen.
Picking up the ball	Visual memory	Pickleball practice with a string attached to a badminton ball to hold the strike position in place. The Rongji group had students look at the model in the teacher’s hand before they practiced picking the ball, and after some time, they were asked to point out the model they had seen before.
Pace combination exercise	Auditory memory	Positions on the field where the pace needs to be changed are marked with markers, and students change their pace according to the markers. The Rongzhi group builds on this foundation by using the “speaker” to play animal sounds, with the teacher controlling the “play-pause” and asking each student what kind of animal they heard during the exercise.
Forehand shot	Auditory attention	Practice hitting a tennis ball suspended in the air. The Unity group uses “speakers” to play music so that when the students hear an accent in the music, they start hitting the ball and stop when they hear the teacher’s whistle.
Pumping	Motor Imitation	Throw the ball up and when it falls to the marked position, perform a draw against the wall. The Rongzhi group builds on this by imitating a simple movement from the teacher before holding the racket and hitting the ball.
Forehand serve	Verbal imitation	Fix the badminton ball at the striking point, students set up the striking position and practice striking the ball. On top of this, the Unity group imitates and repeats the statements or words spoken by the teacher.
Pace + stroke exercise	Object permanence	Following the teacher’s route, run to the marked position, then the supporting teacher throws the ball and the students hit the ball over the net. The Unity Group requires the teacher to ask the students “whether the size of the badminton in the distance is the same as the one in front of them” or “whether there is any change” after the students hit the ball over the net.
Dribbling with racket	Concrete concept learning	The teacher asks the students to place the ball on the racket and run quickly to carry the ball to the finish line. The Rongzhi group requires the teacher to hold red and green markers in a “traffic light,” holding up the green as the students run quickly and stop immediately when the red is held up. Badminton dropped students, will pick up the ball placed on the racket surface. Until the end.
Backhand serve	Abstract concept learning	The ball carrier places the ball on the front side of the body, with the center of gravity on the supporting leg, and the racket carrier leads the racket to hit the ball. In the Unity group, 2 numbered zones are marked on the court and students are asked to serve the ball to the “maximum” or “minimum” zone.

### 2.3 Cognitive ability test program

This cognitive ability test for students with intellectual disabilities uses the Harbin City Special Education School Students’ Personal Growth Test Platform, using the system’s visual, auditory, imitation, object constancy, and conceptual learning indicators for this test.

**Figure 1:**
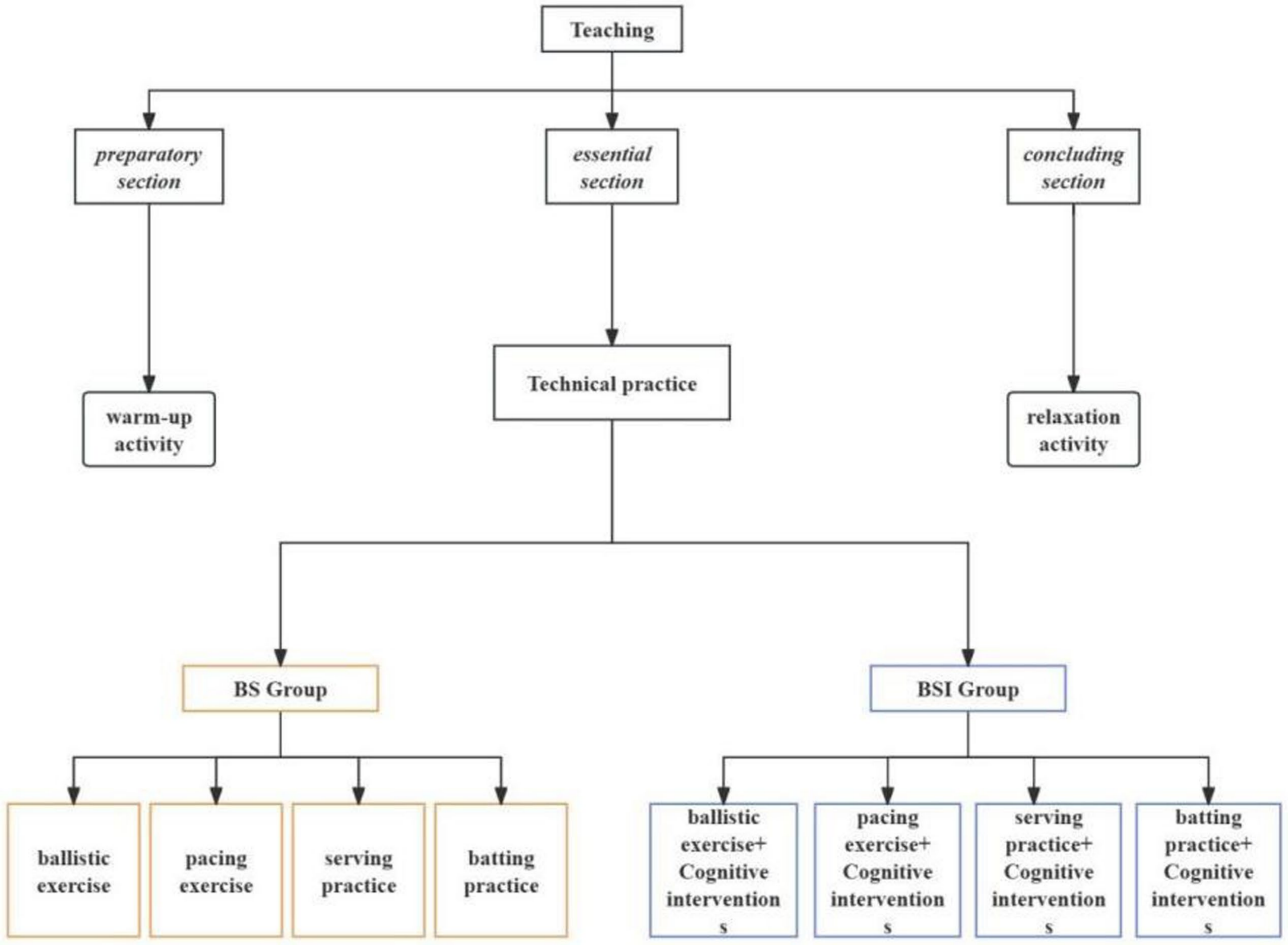
Experimental design and implementation process of this study.

The test was divided into a pre-test and a post-test; in the preparation phase of the assessment test, the assessment selected a quiet and closed classroom, the assessor logged into the system, and the items needed for the test were prepared to ensure that the content of the test subjects can understand and comply with the test requirements. After starting the test, the tester operates according to the content of the test and uses the scoring criteria under its relevant content as the basis for the subject’s score, making a good record of the score. The main contents of the test are attention, memory, imitation, object constancy, and concept learning, as shown in Figure 1.

For example, to illustrate the specific operation of action imitation, the evaluator first asks if children are not familiar with a single body language, such as “two fingers aligned with each other and then fingers crossed” for children to imitate. If the child is unable to complete the task, the assessor then gives the child a familiar single-body movement, such as the “two fingers crossed” movement, for the child to imitate. (Note: If the child is asked to look at a familiar movement and cannot perform it, the child cannot be tested again with the same movement; if the child is asked to perform an unfamiliar movement and cannot perform it, the evaluator can still use the same movement to demonstrate it, and the main point of the evaluation is whether or not the child can learn the movement he/she has just performed.) Scoring criteria: 0 points for the subject cannot reproduce (make) familiar single-limb movements, 1 point can reproduce (make) familiar single-limb movements, 2 points can reproduce (make) unfamiliar single limb coherent movements (Garn and Webster, 2018; Liu et al., 2024).

### 2.4 Data analysis

The collected data were entered into Excel software, and the experimental data were processed and analyzed by SPSS27.0 software. Pre-experimental data were analyzed using one-way ANOVA, with or without differences between the groups in the items tested. Data were collected after the completion of the 12-week intervention experiment, and data before and after the experiment were analyzed by paired-sample t-tests for each group; incremental comparisons between groups were analyzed by covariance tests. In the statistical analysis, *p* < 0.05 was considered a significant difference, and *p* < 0.01 was considered a highly significant difference.

## 3 Results

### 3.1 Pre-intervention cognitive ability test results for students with intellectual disabilities

Table 3 compares cognitive ability outcome data of the badminton physical intelligence group, badminton group, and control group before intervention. The dimensions of visual, auditory, imitation, conceptual, and object constancy were tested through the cognitive ability testing platform in special education schools. Using one-way ANOVA, it was concluded that there was no significant difference between the cognitive ability indicators of the pre-intervention groups (*p* ≥ 0.05).

**Table 3:** Comparison of cognitive abilities between groups before the intervention.

Test items	BSI	BS	CON	*F*	*P*
Visual attention	5.33 ± 1.50	5.13 ± 1.64	6.11 ± 2.26	0.695	0.509
Auditory attention	2.44 ± 0.88	3.00 ± 1.07	2.22 ± 1.92	0.705	0.504
Visual memory	5.44 ± 2.55	6.25 ± 2.43	6.44 ± 3.21	0.328	0.724
Auditory memory	4.00 ± 2.00	4.00 ± 1.20	5.77 ± 2.49	2.336	0.119
Motor imitation	4.00 ± 1.00	4.37 ± 1.30	5.00 ± 1.12	1.766	0.194
Verbal imitation	2.11 ± 1.36	2.50 ± 0.76	2.67 ± 1.58	0.431	0.655
Object constancy	4.0 ± 1.22	4.13 ± 1.55	4.55 ± 1.51	0.371	0.694
Concrete concepts	9.55 ± 2.70	9.50 ± 3.16	10.44 ± 5.20	0.165	0.849
Abstract concepts	6.33 ± 2.74	8.63 ± 2.50	8.22 ± 3.63	1.434	0.259

*Indicates a significant difference at *p* < 0.05.

### 3.2 Results of basic motor skills test for students with intellectual disabilities before intervention

Table 4 shows the results of basic motor skill data testing in the pre-intervention badminton physical intelligence group, badminton group, and control group. The motor skill test used the TGMD-2 scale, and the test items included displacement movement (running, front slide, one-legged jump, front straddle jump, standing long jump, and side slide) and object control (*in situ* racket, hitting a stationary ball, catching a ball with both hands, kicking a ball, overhand throwing, and ground ball). A one-way analysis of variance (ANOVA) yielded no significant differences in the basic motor skill indicators among the three groups before the intervention (*p* ≥ 0.05).

**Table 4:** Comparison of motor skills between groups before the intervention.

Test items	BSI	BS	CON	*F*	*P*
Run	2.44 ± 1.67	2.25 ± 1.39	1.89 ± 2.32	0.209	0.813
Forward sliding step	3.11 ± 1.36	1.88 ± 0.99	3.22 ± 2.54	1.458	0.253
Single-legged jump	3.78 ± 1.86	3.88 ± 2.03	2.44 ± 2.13	1.394	0.268
Front straddle jump	1.56 ± 1.13	2.00 ± 0.76	0.78 ± 1.39	2.537	0.101
Standing long jump	2.33 ± 1.58	2.50 ± 1.51	2.00 ± 1.12	0.279	0.759
Side slide	3.78 ± 1.92	2.50 ± 0.76	3.75 ± 1.39	1.535	0.237
Fixed ball	2.44 ± 1.13	2.50 ± 1.07	1.67 ± 1.12	1.555	0.233
Slap the ball in place	3.11 ± 1.05	3.63 ± 0.74	3.78 ± 1.64	0.731	0.492
Catch with both hands	2.67 ± 1.32	2.63 ± 1.06	2.89 ± 1.17	0.123	0.885
Kick	3.33 ± 1.22	3.38 ± 0.74	2.44 ± 2.13	1.074	0.358
Overhand throw	2.33 ± 1.00	2.50 ± 0.93	3.11 ± 2.62	0.500	0.613
Ground ball	2.00 ± 0.87	1.50 ± 0.53	2.33 ± 2.00	0.851	0.440
Total points	32.89 ± 11.22	32.38 ± 9.44	31.67 ± 12.52	0.027	0.973

*Indicates a significant difference at *P* < 0.05.

### 3.3 Post-intervention cognitive test results for students with intellectual disabilities

#### 3.3.1 Visual ability test results

Comparison of the results of BSI, BS, and CON in the test of cognitive ability in visual ability before and after the intervention, the test of visual ability includes visual attention and visual memory, the data before and after the intervention were analyzed by paired samples t-test, and the results showed that: (1) in visual attention: there was no significant difference in the data of CON before and after the intervention; BSI had a very significant difference in the comparison between the situation before and after the intervention; BS had a significant difference. (2) In terms of visual memory, the BSI has a very significant difference, the BS has a significant difference, and the CON has no significant difference, as shown in Table 5.

**Table 5:** Comparison of visual ability between groups before and after intervention.

Test items	Group	Pre-intervention	Post-intervention	Mean difference	*T*	*P*
Visual attention	BSI	5.33 ± 1.50	7.11 ± 1.05	1.78	−4.438	0.002**
	BS	5.13 ± 1.64	6.12 ± 0.83	1.00	−4.320	0.043*
	CON	6.11 ± 2.26	6.00 ± 1.80	0.22	−0.800	0.447
Visual memory	BSI	5.44 ± 2.55	8.11 ± 1.45	2.00	−6.000	0.000**
	BS	6.25 ± 2.43	7.50 ± 1.69	1.25	−2.376	0.049*
	CON	6.44 ± 3.21	5.89 ± 3.44	−0.56	1.474	0.179

*Indicates *P* < 0.05, a significant difference; ** indicates *P* < 0.01, a highly significant difference.

As shown in Table 6, for comparing the incremental visual ability of BSI, BS, and CON before and after the intervention, the incremental changes among the groups were analyzed by analysis of covariance. (2) Visual attention: there was a significant difference in incremental change between BSI and BS; the mean value of incremental change in CON was lower than that of BSI and was significantly different; there was also a significant difference in incremental change between BS and CON. (3) Visual memory: the incremental mean changes of BSI and BS, respectively, compared to CON, were significantly different; BSI compared to BS was not significantly different.

**Table 6:** Comparison of incremental visual ability between groups before and after intervention.

Test items	BSI	BS	CON	*F*	*P*
Visual attention	1.78 ± 1.20a	1.00 ± 1.31b	0.22 ± 0.83c	7.905	0.003**
Visual memory	2.00 ± 1.00a	1.25 ± 1.49a	−0.56 ± 1.13b	15.234	0.000**

Data labeled with the same letter mark or no letter mark represent no significant difference in data between groups (*P* ≥ 0.05); those labeled with different letter marks represent significant differences between groups (*p* < 0.05); * indicates *p* < 0.05 and ** indicates *p* < 0.01.

#### 3.3.2 Auditory ability test results

Comparison of the results of BSI, BS, and CON in the cognitive ability test of auditory ability before and after the intervention. The auditory ability test includes auditory attention and auditory memory, and the data of the test before and after the intervention were analyzed through the paired samples t-test, and the results showed that: (1) In terms of auditory attention, the mean value of the pre-intervention of the BSI was smaller than the post-intervention, with a very significant difference; and there was no significant difference between the BS and the CON. (2) Regarding auditory memory, the pre-intervention mean of BSI was smaller than the post-intervention, with a highly significant difference; there was no significant difference between BS and CON, as shown in Table 7.

**Table 7:** Comparison of auditory ability between groups before and after intervention.

Test items	Group	Pre-intervention	Post-intervention	Mean difference	*T*	*P*
Auditory attention	BSI	2.44 ± 0.88	4.00 ± 1.32	1.55	−4.603	0.002**
	BS	3.00 ± 1.07	3.25 ± 1.03	0.25	−1.000	0.351
	CON	2.22 ± 1.92	2.33 ± 1.94	0.11	−0.263	0.799
Auditory memory	BSI	4.00 ± 2.00	5.55 ± 2.60	1.55	−5.292	0.001**
	BS	4.00 ± 1.20	4.38 ± 1.06	0.38	−2.049	0.080
	CON	5.77 ± 2.49	6.22 ± 1.68	0.44	−1.512	0.169

*Indicates *P* < 0.05, a significant difference; ** indicates *P* < 0.01, a highly significant difference.

As shown in Table 8, for the comparison of incremental changes in the auditory ability of BSI, BS, and CON before and after the intervention, the incremental changes among groups were analyzed by analysis of covariance, which showed that (1) the incremental changes of the three groups were significantly different in terms of auditory attention; and very significantly different in terms of auditory memory. (2) Auditory attention: the mean incremental value of BSI compared with BS was greater than that of CON, which was a significant difference; for other inter-group comparisons, there was no significant difference. (3) Auditory memory: the incremental means of BS and CON were lower than that of BSI compared to BSI, respectively, both of which were significantly different; there was a significant difference in the incremental value of BS compared to CON.

**Table 8:** Comparison of incremental auditory ability between groups before and after intervention.

Test items	BSI	BS	CON	*F*	*P*
Auditory attention	1.55 ± 1.01a	0.25 ± 0.71b	0.11 ± 1.27b	5.312	0.013*
Auditory memory	1.55 ± 0.88a	0.38 ± 0.52b	0.44 ± 0.88b	6.557	0.006**

Data labeled with the same letter mark or no letter mark represent no significant difference in data between groups (*P* ≥ 0.05); those labeled with different letter marks represent significant differences between groups (*p* < 0.05); * indicates *p* < 0.05 and ** indicates *p* < 0.01.

#### 3.3.3 Imitation ability test results

Comparison of the results of the test of imitation ability in cognitive intervention before and after the intervention of BSI, BS, and CON. Imitation ability includes action imitation and verbal imitation, and the data tested before and after the intervention were analyzed by the paired samples *t*-test; CON, on the other hand, had no significant difference. (2) In verbal imitation, the pre-intervention mean of BSI was smaller than the post-intervention, with a highly significant difference; BS and CON had no significant difference, as shown in Table 9.

**Table 9:** Comparison of imitation ability between groups before and after intervention.

Test items	Group	Pre-intervention	Post-intervention	Mean difference	*T*	*P*
Action figure	BSI	4.00 ± 1.00	5.55 ± 0.53	1.55	−6.424	0.001**
	BS	4.37 ± 1.30	5.50 ± 0.53	0.88	−2.966	0.021*
	CON	5.00 ± 1.12	4.77 ± 1.09	0.22	0.800	0.447
Language imitation	BSI	2.11 ± 1.36	3.33 ± 0.83	1.22	−4.400	0.002**
	BS	2.50 ± 0.76	2.75 ± 0.89	0.25	−1.528	0.170
	CON	2.67 ± 1.58	3.11 ± 1.27	0.44	−1.315	0.225

*Indicates *P* < 0.05, a significant difference; ** indicates *P* < 0.01, a highly significant difference.

As shown in Table 10, for the comparison of the increment of imitation ability of BSI, BS, and CON before and after the intervention, the increment was analyzed by analysis of covariance (ANCOVA) between the groups, and the results showed that (1) BSI, BS, and CON had a highly significant difference in action imitation; and in verbal imitation, BSI had a highly significant difference. (2) Action imitation: the incremental mean values of BSI and BS were higher than that of CON, respectively, and were significantly different; BSI was not significantly different from CON. (3) Verbal imitation: the incremental means of BS and CON were lower than those of BSI and were significantly different; the incremental changes between BS and CON, on the other hand, were not significantly different.

**Table 10:** Comparison of incremental imitation ability between groups before and after intervention.

Test items	BSI	BS	CON	*F*	*P*
Action figure	1.55 ± 0.73a	0.88 ± 0.83a	0.22 ± 0.83b	8.154	0.002**
Language imitation	1.22 ± 0.83a	0.25 ± 0.46b	0.44 ± 1.01b	7.774	0.004**

Data labeled with the same letter mark or no letter mark represent no significant difference in data between groups (*P* ≥ 0.05); those labeled with different letter marks represent significant differences between groups (*p* < 0.05); * indicates *p* < 0.05 and ** indicates *p* < 0.01.

#### 3.3.4 Conceptual learning ability test results

Comparison of the test results of conceptual learning ability of BSI, BS, and CON in cognitive intervention before and after the intervention. Conceptual learning ability includes concrete and abstract concepts, and the data tested before and after the intervention were analyzed by paired samples *t*-test, and the results showed that (1) in terms of concrete concepts, the mean value of the BSI pre-intervention measurements was smaller than that after the intervention, and there was a very significant difference, and there was no statistically significant difference in the data measured by the BS and CON Before and after the measured data, there was no statistical difference. (2) In terms of abstract concepts, there was a non-significant difference in BSI; there was no statistically significant difference in BS and CON, as shown in Table 11.

**Table 11:** Comparison of learning ability between groups before and after intervention.

Test items	Group	Pre-intervention	Post-intervention	Mean difference	*T*	*P*
Specific concepts	BSI	9.55 ± 2.70	11.66 ± 4.06	2.11	−3.591	0.007*
	BS	9.50 ± 3.16	9.87 ± 3.18	0.38	−1.426	0.197
	CON	10.44 ± 5.20	10.00 ± 5.15	−0.44	0.883	0.403
Abstract concepts	BSI	6.33 ± 2.74	9.11 ± 3.89	2.78	−5.330	0.000**
	BS	8.63 ± 2.50	9.00 ± 2.14	0.38	−2.049	0.080
	CON	8.22 ± 3.63	7.67 ± 3.74	−0.55	1.048	0.325

*Indicates *P* < 0.05, a significant difference; **indicates *P* < 0.01, a highly significant difference.

As shown in Table 12, for the comparison of incremental concept learning ability of BSI, BS, and CON before and after the intervention, the incremental increase among the groups was analyzed by analysis of covariance, and the results showed that (1) the three groups had a highly significant difference in terms of concrete concepts and abstract concepts. (2) Concrete concepts: BSI incremental mean is higher than CON, which is a significant difference; CON compared to BS in terms of the change of incremental mean is not a significant difference. (3) Abstract concepts: BSI incremental mean change is higher than BS and CON and is significantly different; BS is not significantly different compared to CON.

**Table 12:** Comparison of incremental learning ability between groups before and after intervention.

Test items	BSI	BS	CON	*F*	*P*
Specific concepts	1.55 ± 0.73a	0.88 ± 0.83a	0.22 ± 0.83b	8.154	0.002**
Abstract concepts	1.22 ± 0.83a	0.25 ± 0.46b	0.44 ± 1.01b	7.774	0.004**

Data labeled with the same letter mark or no letter mark represent no significant difference in data between groups (*P* ≥ 0.05); those labeled with different letter marks represent significant differences between groups (*p* < 0.05); * indicates *p* < 0.05 and ** indicates *p* < 0.01.

#### 3.3.5 Object constancy test results

Comparison of the results of the BSI, BS, and CON in the object constancy test in the cognitive intervention before and after the intervention The data tested before and after the intervention were analyzed using a paired samples t-test, which showed that the data analyzed as measured by the BSI were significantly different, whereas there was no significant difference between the BS and the CON, as shown in Table 13.

**Table 13:** Comparison of object constancy in each group before and after the intervention.

Test items	Group	Pre-intervention	Post-intervention	Mean difference	*T*	*P*
Object constancy	BSI	4.00 ± 1.2	5.33 ± 0.7	1.33	−5.657	0.001**
	BS	4.13 ± 1.55	4.50 ± 0.93	0.38	−1.426	0.197
	CON	4.55 ± 1.51	4.33 ± 1.73	−0.22	0.286	0.782

*Indicates *P* < 0.05, a significant difference; ** indicates *P* < 0.01, a highly significant difference.

## 4 Discussion

The cognitive ability of students with intellectual disabilities is an important indicator of intelligence, as their own deficiencies cause them to lag behind normal students in cognitive level, which has a serious impact on their intelligence (Alnahdi, 2019). Therefore, many experts and scholars have used various interventions to improve the cognitive abilities of people with intellectual disabilities and to help them participate in social life in the future (Hollis et al., 2017; Frances et al., 2022). Previous studies have concluded and proved that sports could improve the cognitive level of the intellectually disabled group, but it is especially necessary to pay attention to the choice of sports and the operation process of teaching skills; simple and mechanical forms of sports activities are not necessarily able to get higher acceptance of the training object, such as running, resistance training, etc. (Ginis et al., 2021). Therefore, some scholars believe that individual small ball sports activities that are rich in fun seem to be more easily accepted by this kind of children’s locks (Chang, 2021).

The deficits in attentional functioning that exist in the mentally handicapped group become one of the obstacles that affect their normal life (Crespi et al., 2010; Shogren et al., 2014). Therefore. The attentional function in this study is mainly tested in terms of visual attention (van der Aa et al., 2023) and auditory attention (Kim et al., 2021) dimensions. Visual attention is a subsystem in the attention system under neuropsychological mechanisms and is one of the research directions in the field of contemporary cognitive psychology and neuroscience (Lu et al., 2023). Auditory attention refers to the stimulation of external sound signals by a person, which causes the neural mechanisms of the brain to make processing (Obleser and Kayser, 2019). Numerous studies have shown that auditory attention reflects the neural development of the brain and plays a crucial role in human cognition (van Ede and Nobre, 2023; Liu et al., 2022). In this study, it was found that there was a significant difference between the pre-and post-intervention means of the physical intelligence group and the badminton group in terms of visual attention (*p* < 0.05); the increment of both groups was higher than that of the control group. Regarding auditory attention, only the physical and intellectual groups had a significant difference (*p* < 0.05). This shows that the teaching intervention program for the badminton physical intelligence group was superior to that of the badminton group. Similar to the previous study, after 8 weeks of badminton practice intervention, it was found that the visual attention level of junior high school students increased significantly in the dimensions of transfer, breadth, stability, and distribution, and it was concluded that the high demands of badminton on visual and auditory attention help stimulate the trainers’ brains. The good effect of the “body-intelligence integration” badminton teaching in this study may be because the varied forms of badminton and the need to analyze the changes in the field of play stimulate an increase in dopamine in the frontal lobe central receptors, which leads to an improvement in neuronal receptor blunting (Protzner et al., 2015; Abdollahipour et al., 2023).

In previous studies on normal children, it was found that the effects of different sports on visual memory were found to have an effect on the student’s visual memory and that visual attention had an interactive effect on the development of visual memory (Maenner et al., 2021; Baio et al., 2018; Liu et al., 2024; Alnahdi, 2019; Abdollahipour et al., 2023; Lind et al., 2019; Benzing et al., 2018; Chan et al., 1893). However, less attention has been paid to special groups; this study found that in visual memory, the BSI and BS groups had a significant effect before and after the experiment; the increment of both groups was higher than that of the control group (*p* < 0.05). In auditory memory, the BSI group had a significant effect on change, and its incremental change was the largest. In badminton, technical movements of the body force, coordination, accuracy, and other requirements must be dominated by the brain of students with intellectually disabled control, cortical nervous system excitation, and inhibition. The sport itself can increase the degree of excitation of the nervous system, and endocrine hormones produced by the movement can also promote the transmission of chemical information in the synapses (Dongmoon and Eun-Sun, 2018). More critically, the integration of intellectual training into badminton through the exercise increases the excitability of the nervous system integration of memory content interventions suitable for students with intellectually disabled, prompting the formation of new neural networks and increasing the plasticity of synapses, resulting in the improvement of their memory (Ludyga et al., 2022; Ludyga et al., 2016).

As a core mechanism of imitation ability as a unique social cognition, previous studies have suggested that the generation of imitation ability is associated with the associative activation of visual and motor representations in imitative behaviors (Web of Science, 2025a). It has been found that imitative ability can lead to gains in language development and motor skill acquisition in exceptional students through imitative training in receptive language and gesture and can activate the imitator’s cortical nerves to speed up the transmission and processing of the received information, resulting in an increase in the functional activity of the mirror nervous system (Hale and Hamilton, 2016). The imitation ability in this study is mainly from two aspects: motor and verbal. In this study, it was found that the BSI group was better than the BS group in terms of improving this ability. Dual-task training combining cognitive and ball games was found to be more effective in improving this ability in this group of children, probably because of the close relationship between the level of activation and activity of the brain’s nervous system (Koch et al., 2018). It has been suggested that imitation is associated with extensive activity in the cerebral cortex, resulting in increased bilateral activity in several motor subcortical areas in the cerebellum, central sulcus, and supplementary motor areas (Al-Hashimi et al., 2015; Moisala et al., 2016).

Numerous previous studies have shown that appropriate exercise improves the quality and efficiency of students’ learning and has a positive effect on their cognitive abilities. In an intervention on object class concepts for children with intellectual disabilities aimed at improving their thinking skills, it was thought that it was possible that the addition of exercise along with separate thinking exercises would have better results, and our study confirms this previous conjecture (Web of Science, 2025b; Moore, 2023). The comparison of means before and after the intervention of the BSI had a significant change in effect, while there was no change in effect for the BS and CON. Increments in the BSI compared to the CON and BS groups, respectively, had a significant difference in effect. CON and BS groups were significantly different compared to each other. It is possible that this result is because through exercise, the body secretes physiological hormones that affect the activity of the nervous system, such as norepinephrine (Pietrelli et al., 2018), dopamine (Petrican et al., 2024), and other physiological hormones (Donato and Kopchick, 2023), which can promote the excitation and activity of the cerebral cortical nerves, so that the energy substance is sufficiently used within the brain, and the inter-neural information transmission is accelerated, improving the concentration and agility of thinking (Janssen et al., 2014).

It can be seen from the above analyses that the improvement of cognitive ability by “physical and intellectual” integration of badminton teaching interventions is significantly better than that of badminton teaching interventions. Therefore, the effective combination of physical education and cognitive intervention is conducive to the overall development of students with intellectual disabilities. After this study, it was found that the cognitive intervention program should be reasonably designed according to the characteristics of the sport.

## 5 Conclusion

This study found that compared with badminton alone, adding cognitive training intervention to badminton can effectively improve the cognitive ability of students with intellectual disabilities in vision, hearing, imitation, concept learning, object constancy and other aspects, and has a better effect on the object control of children in this group, which may be because the interesting nature of badminton itself amplifying the effect of cognitive training. Based on this, in the special education for such children, teachers can consider adding cognitive training to badminton to improve the well-being of special children.

## Data Availability

The original contributions presented in the study are included in the article/supplementary material, further inquiries can be directed to the corresponding author/s.
